# Sodium and potassium intakes and adiposity among Iranian pre-adolescents and adolescents: a cross-sectional study

**DOI:** 10.1186/s12937-022-00776-y

**Published:** 2022-04-28

**Authors:** Sahar Golpour-Hamedani, Nahid Rafie, Makan Pourmasoumi, Sayyed Morteza Safavi, Noushin Mohammadifard

**Affiliations:** 1 Food Security Research Center, Isfahan University of Medical Sciences, Isfahan, Iran; 2 Department of Clinical Nutrition, School of Nutrition and Food Sciences, Isfahan University of Medical Sciences, Isfahan, Iran; 3 Gastrointestinal and Liver Diseases Research Center, Guilan University of Medical Sciences, Rasht, Iran; 4 Isfahan Cardiovascular Research Center, Cardiovascular Research Institute, Isfahan University of Medical Sciences, Isfahan, Iran

**Keywords:** Sodium, Potassium, 24-h urine, Obesity, Pre-adolescents, Adolescents

## Abstract

**Background:**

High sodium and low potassium intakes are associated with the early development of chronic diseases (e.g., hypertension, obesity). Taking into account the limited data on sodium and potassium intakes by 24-h excretion in urine in pre-adolescents and adolescents, we wished to determine baseline salt intake in Iranian subjects aged 11–18 years.

**Methods:**

This was an observational study involving 374 pre-adolescents and adolescents (154 boys and 220 girls). Sodium and potassium intakes were ascertained by measuring sodium and potassium excretion in urine over 24 h. Creatinine level was used to validate the completeness of the urine collections. The association between sodium and potassium intake and adiposity was determined based on body fat percentage.

**Results:**

The mean 24-h urine sodium concentration was 3130 ± 2200 mg/day, equal to 7.961 ± 5.596 g/day salt intake. Approximately half of the study participants exceeded the upper limit of Na intake. The mean potassium intake was estimated 1480 ± 1050 mg/day. There was a positive association between urinary sodium excretion and adiposity in crude (OR 1.79; 95% CI: 1.08—2.74) and full adjusted model (OR: 3.15; 95% CI: 2.28–4.63). Also, in subsample analysis, there was a positive correlation between urinary sodium and adiposity in both pre-adolescents (OR: 2.71; 95% CI: 2.29—3.93) and adolescents (OR: 3.55; 95% CI: 2.17—4.74). However, no significant association was found between 24-h urinary potassium and adiposity.

**Conclusion:**

Sodium intake, as estimated by 24-h urinary excretion, was higher than recommended and it was positively associated with adiposity. Also, this study reported low compliance of potassium intake recommendations in 11–18 years’ Iranian pre-adolescents and adolescents. Health promotion interventions are needed in order to broaden public awareness of high sodium intake and potassium inadequacy to reduce chronic diseases.

## Introduction

Excessive sodium (Na) and inadequate potassium (K) intakes are associated with an increased risk of high blood pressure (BP), which is the primary risk factor for cardiovascular diseases (CVDs) [1, 2]. Recent evidence shows that salt plays a key role in regulating BP in children [1, 3]. Subjects with high BP at an early age are more likely to develop hypertension in the future [4]. Also, high Na and low K intakes have detrimental effects on obesity, and both are associated with other chronic diseases and premature death [1, 5]. There is also evidence on Na intake in relation to body weight in children and adolescents independent of energy intake [6]. In addition, it has been found that K intake slows the harmful effects of Na, although this has only been shown in relation to BP [7]. There are few epidemiologic studies indicating a positive correlation between high Na and low K intakes and adiposity measures [8, 9].

Studies on Na and K intakes in young children are relatively few and the global data in this regard are mainly limited to Europe and North America [1]. Likewise, available information about Iranian children's Na and K intake is very limited. One study of Iranian children aged 3–10 years indicated a high intake of Na in this population (2017 mg/day), assessed using a spot urine sample [10].

According to the public health priorities, reducing salt intake is one of the easiest, most efficient, and cost-effective ways to control the burden of health disorders [11]. Therefore, determining Na intake among children using standard measurements is considered an important first step in the implementation of health programs. The use of dietary surveys and databases detailing food composition can over-or underestimate the actual intake [12, 13]. The best method of estimating Na and K intakes is the measurement of its excretion over 24 h, which is considered the gold standard to assess dietary intake [14].

To the best of our knowledge, there is no data on 24-h urinary excretion of Na and K in Iranian pre-adolescents and adolescents. Thus, we aimed to evaluate and provide information about consumption levels of Na and K and their relation to adiposity, measured by 24-h urinary excretion in a sample of Iranian individuals aged 11–18 years old.

## Materials and methods

### Study design and participants

This cross-sectional study was conducted on healthy individuals aged 11–18 years in Isfahan city, Iran. Using the cluster random sampling method, participants were selected from 13 schools of 4 different districts. Within participating schools, invitations were sent to the parents. All parents/students who agreed to participate in the study signed informed a written consent. Participants with any acute or chronic diseases or on any medication or special diet were excluded from the study.

Of 456 participants who received the 24 h urine containers, 50 were not returned. Samples with volumes of less than 500 ml, more than one missed voiding, and/ or 24-h creatinine excretion less than 0.1 mg/ kg body weight were discarded [15]. Also, participants who stated that they had missed any urine collections were excluded from the study.

Also, some discarded urine samples due to volumes of less than 500 ml were excluded (*n* = 32). Thus, 374 participants had complete and valid urinary samples and questionnaires and were included in the final analysis (response rate 82%). The study protocol was approved by the Research Ethics Committee of Isfahan University of Medical Sciences (IR. mui.rec.1394.3/ 294).

### Twenty-four h urine collection

Participants and parents were provided with 24-h urine collection containers, along with verbal and written instructions on how to complete the collection. It was emphasized that no change in dietary intake during the day of collection was allowed. The urine collection was completed over the weekends from Friday to Saturday, during a 24-h period. Each participant was provided with a 2.5-L polypropylene container used for the collection of a 24-h urine sample. All participants were instructed to initiate the collection by emptying their bladder, discarding the first urination after waking on Friday morning, and continuing collection until the morning of the following day. To assist urine collection, an additional 500 ml plastic cup was provided. All participants were asked to keep the container in a cool and dry place and samples were immediately transferred to the laboratory the next day for further analysis in order to prevent microbial degradation. If a person was unable to deliver the urine sample for any reason, the sample was retrieved from their home by a member of the research team.

Measured parameters included 24-h urine volume, Na, K, and creatinine levels. Na and K were estimated by the ion-selective electrode method and urine creatinine was measured using the Jaffe reaction method [16].

### Anthropometric assessment

Anthropometric parameters, including weight and height, were measured using the following standard protocols with light clothing and without shoes. Weight, body mass index (BMI), percentage body fat (PBF), and lean body mass (LBM) were all measured using an Omron digital scale (BF511, Kyoto, Japan). Body fat percentage ≥ 25% in boys and 35% in girls was defined as being at risk of adiposity [17].

### Assessment of other variables

Additional socio-demographic information, including age, sex, parents’ educational level, household income, and past medical history were obtained from a self-administered questionnaire completed by participants and/or their parents/guardians. Also, children's physical activity was assessed through a self-administrated7-day recall questionnaire (PAQ) with high validity and moderate reliability [18], which was also, fulfilled by participants themselves and/or their parents/guardians. PAQ assesses participants in different physical activities in the school, after school, evenings, and weekends with 9 items, each scored on a 5-point scale. A value from 1 to 5 is obtained for each of the 9 items and used in the physical activity composite score and the mean of these scores becomes the final PAQ activity score. A score of 1 indicates low physical activity, a score of 2–4 indicates moderate physical activity, and a score of 5 indicates high physical activity.

### Statistical analysis

Descriptive statistics (mean values and standard deviations for continuous variables or numbers and percentages for categorical variables) were used to describe participants' characteristics. We considered participants aged 11–14 years as pre-adolescents and 15–18 years as adolescents. One-way ANOVA and chi-square tests were used where appropriate. Multiple logistic regression models were used to assess the association between 24-h urinary Na and K excretion with PBF. The unadjusted and adjusted models (age, sex, parental educational level, household income, physical activity) are presented. To assess whether the association between 24-h urinary Na and K excretion with adiposity was independent of energy and sugar-sweetened beverages (SSBs) intake (including carbonated soft drinks, soda, squashes, and industrial fruit drinks), additional models were constructed involving these covariates. In all multiple models, the first tertile of Na and K excretion was considered as the reference. Analyses were completed using SPSS for Windows (version 18, SPSS Inc., Chicago, IL., USA), and a P-value < 0.05 was considered statistically significant.

## Results

Table 1 shows the main characteristics of the study participants and information on urinary excretion. Of 374 participants, 58.8% (*n* = 220) were girls (mean age: 14.4 ± 2.02 years). The mean 24-h urine Na concentration was 3130 ± 2200 mg/ day, equal to 7.961 ± 5.596 g/day salt intake. Approximately half of the study participants exceeded the upper limit of Na intake. The mean K intake was estimated 1480 ± 1050 mg/day, and the excretion of 24-h urine K in girls and boys was 1320 ± 890 and 1700 ± 1200 mg/day. The majority of participants received less than recommended levels.

**Table 1:** Personal characteristics of the study population and twenty-four-hour urine data by sex

Variables		Total *(n* = 374)	Boys (*n* = 154)	Girls (*n* = 220)	*P*-value
Age (year)		14.4 ± 2.09	13.8 ± 1.96	14.8 ± 2.10	< 0.001
Body fat (%)		24.99 ± 9.65	20.11 ± 9.04	28.30 ± 8.60	< 0.001
Adiposity n (%)		93 (24.9)	43 (11.5)	50 (13.4)	0.16
Weight (kg)		53.2 (14.20)	54.20 (16.90)	52.57 (11.98)	0.27
24-h urinary sodium (mg/d)		3130 ± 2200	3540 ± 2440	2840 ± 1980	0.004
Salt intake (gr)		7.961 ± 5.596	9.004 ± 6.206	7.223 ± 5.036	0.004
24-h urinary potassium (mg/d)		1480 ± 1050	1700 ± 1200	1320 ± 890	0.001
24-h creatinine (mg/kg)		0.14 ± 0.08	0.15 ± 0.08	0.12 ± 0.07	0.001
24-h volume (ml)		920 ± 380	950 ± 380	900 ± 380	0.18
Energy intake (Kcal)		1676 ± 301	1759 ± 327	1619 ± 267	< 0.001
SSB intake (ml)		43.73 ± 42.05	44.70 ± 41.49	43.05 ± 42.51	0.71
Physical activity n (%)	Low	136 (36.36)	39 (25.32)	97 (44.09)	< 0.001
	Medium	238 (63.64)	115 (74.68)	123 (55.91)	
	High	-	-	-	

Odds ratios (ORs) for adiposity across tertiles of Na and K excretion are provided in Table 2 and 3. In the total sample of 11–18-year-olds, those in the highest tertile of Na excretion had higher odds of adiposity based on body fat percentage as compared with those in the lowest category in the crude model (OR: 1.79; 95% CI: 1.08–2.74). The association remained significant after adjusting for potential confounders including age, sex, family income, parents educational level, physical activity, calorie and SSBs intake (OR: 3.15; 95% CI: 2.28- 4.63).

**Table 2:** Odds ratios and 95% CI for adiposity according to tertiles of 24-h urinary sodium among Iranian children and adolescents aged 11–18 years, Isfahan, Iran

Tertiles of urinary sodium			
**Total**	**T1**	**T2**	**T3**
Crude	1	1.31 (0.83–2.18)	1.79 (1.08–2.74)
Model I	1	1.74 (0.75–2.88)	1.94 (1.23–2.89)
Model II	1	2.34 (1.05–3.67)	2.92 (2.01–3.79)
Model III	1	2.63 (1.04–3.61)	3.15 (2.28–4.63)
**Age groups**			
**Children (11–14 years)**			
Crude	1	1.77 (0.81- 3.91)	1.53 (1.09- 3.86)
Model I	1	2.28 (1.42- 4.09)	2.99 (2.59- 3.91)
Model II	1	2.18 (1.83- 4.51)	2.27 (2.56- 3.65)
Model III	1	2.26 (1.01–4.84)	2.71 (2.29- 3.93)
**Adolescents (15–18 years)**			
Crude	1	1.87 (0.88- 3.12)	1.89 (1.21- 3.72)
Model I	1	2.17 (1.71- 3.16)	2.20 (3.11- 4.49)
Model II	1	2.79 (1.27- 4.93)	2.12 (3.03- 4.41)
Model III	1	2.74 (0.98–4.06)	3.55 (2.17- 4.74)

Model I: adjusted for age and sex

Model II: further adjusted for parents’ educational level, household income and physical activity

Model III: more adjustment for SSB and total calorie intake

**Table 3:** Odds ratios and 95% CI for adiposity according to tertiles of 24-h urinary potassium among Iranian children and adolescents aged 11–18 years, Isfahan, Iran

Tertiles of urinary potassium			
**Total**	**T1**	**T2**	**T3**
Crude	1	1.13 (0.50–2.59)	1.38 (0.52–3.68)
Model I	1	1.14 (0.50–2.62)	1.37 (0.51–3.68)
Model II	1	0.78 (0.32–1.92)	0.94 (0.32–2.79)
Model III	1	0.69 (0.28–1.72)	0.57 (0.18–1.81)
**Age groups**			
**Children (11–14 years)**			
Crude	1	2.06 (0.55- 3.97)	5.16 (2.98- 9.45)
Model I	1	1.59 (0.72- 3.48)	4.67 (2.85- 9.89)
Model II	1	1.45 (0.84–3.56)	2.63 (1.45- 5.65)
Model III	1	0.91 (0.66- 1.84)	0.56 (0.18- 1.49)
**Adolescents (15–18 years)**			
Crude	1	1.81 (0.65- 3.34)	5.11 (2.74- 9.54)
Model I	1	1.67 (0.96- 3.42)	4.51 (2.95- 9.69)
Model II	1	1.52 (0.75–3.95)	2.48 (1.57- 5.39)
Model III	1	0.99 (0.35- 1.96)	0.49 (0.17- 1.95)

Model I: adjusted for age and sex

Model II: further adjusted for parents’ educational level, household income and physical activity

Model III: more adjustment for SSB and total calorie intake

Likewise, in the subgroup analysis on pre-adolescents aged 11–14 years and adolescents aged 15–18 years, both pre-adolescents and adolescents in the highest tertile of Na excretion had higher odds of adiposity compared with those in the lowest category (OR: 2.71; 95% CI: 2.29–3.93) and (OR: 3.55; 95% CI 2.17- 4.74), respectively. Also, after adjusting for confounders, there was a negative but not statistically significant association between urinary potassium excretion and body fat percentage in both pre-adolescents and adolescents.

## Discussion

As far as we know, this is the first study in Iran to evaluate Na and K intakes in a sample of pre-adolescents and adolescents aged 11- 18 years using 24-h urine excretion. The results demonstrated poor adherence to Na and K intake recommendations as 46.1% and 80.5% of participants did not meet the recommendations for Na and K intakes, respectively. Also, there was a positive association between Na excretion and adiposity among study participants.

Data related to Na intake in Iran are limited and there is no data based on 24-h urinary samples especially in pre-adolescents and adolescents. A study conducted on Iranian children aged 3- 10 years showed that the mean urinary sodium excretion was 177.17 mmol/day [10]. However, the mentioned study used spot urine samples to estimate the intakes. Similar results were found in other Asian countries using urine samples. In a study of 6–10 years old Lebanese children [19], the estimated mean Na intake was 96.57 ± 61.67 mmol/day. Also, a study in Japan on 3-year-old children found that urinary Na concentration was 140 ± 67 mmol/d [20]. Studies in European adolescents including Italy, Spain, England, and Germany have yielded comparable results [21–24]. Generally, Na excretion in boys was greater than in girls, which is consistent with our results. This may be due to the higher food consumption levels by boys.

There are also studies evaluating Na intake in children using dietary questionnaires. In Korea, mean Na intake was 4100 mg/d [25] and in Australian 14–16 years old children, mean dietary Na intake was reported to be 2480 mg/d [26]. In the present study, the mean Na intake assessed by a food frequency questionnaire was 269 l mg/d, which is lower than Korean and Australian reports in children.

In this study, the mean K excretion was below WHO recommendations, which is consistent with some other studies reporting low K intake in pre-adolescents and adolescents. Among Dutch children aged 5- 17 years, mean K excretion was 1708.67 mg/d [27] and among Portuguese children, only 6.1% of boys and 7.1% of girls met the recommendations for K intake [28].

Furthermore, this study for the first time shows a positive correlation between PBF and 24 h urinary Na excretion in Iranian pre-adolescents and adolescents, which is consistent with previous studies suggesting a higher likelihood of obesity in children with high Na intake [6, 29]. Among Australian children aged 4–12 years, with an additional 17 mmol/d of Na, the risk of being overweight/obese or abdominally obese increased by 23% and 15%, respectively[30]. They revealed that the potential adipogenic effect of Na is associated with the total body weight and is not specific to central fat distribution. Similar to our study, in a longitudinal study conducted on German children and adolescents aged 3–18 years, a positive association was found between Na intake and BMI z score [31]. It shows that the effects of Na on weight are not affected by puberty and hormones, suggesting that Na can cause obesity in all ages and both genders [31].

Studies on the association between K intake and risk of obesity have led to different results. While Murakami et al. [32] in their study on Japanese young women showed a reciprocal relationship between 24 h urine K and abdominal obesity, studies performed by Shin et al. [33] and Lee et al. [34] showed that receiving K had no effect on obesity. Also, the results of a pooled analysis and systematic review done by Cai X et al. [35] showed that high K intake could not reduce the risk of obesity, while serum K was associated with obesity. In the present study, K showed only an inverse association with adiposity after adjusting the effect of 24 h urine Na, indicating that high K, along with a reduction in dietary Na intake, can reduce the risk of obesity. Most studies in this field have been conducted over the past five years, indicating that K effect on obesity is a new topic, and more robust studies with better design are warranted [34].

Several mechanisms may explain the association of Na and K intakes with obesity risk. First, diets high in Na and low in K are often high in energy and therefore may promote weight gain [26]. Besides, through the effects on thirst, a salty diet may cause a greater consumption of SSBs, which are associated with weight gain [26, 30, 36]. However, after adjusting for intake of energy and SSBs, we found that the association between Na intake and adiposity remained significant, suggesting that other mechanisms may be involved [30, 37]. Experimental studies suggest that higher Na intake is associated with increased lipogenic activity and the formation of adipocyte tissue [38]. Moreover, the uptake of glucose and its conversion to lipids within adipocytes increased in mice as their Na intake increased [38, 39]. The mechanism of dietary K intake in relation to obesity risk is less well- understood. It has been suggested that obesity is associated with K channel function [40, 41]. K can influence carbohydrate accumulation and glucose homeostasis [42, 43] and can lay an important role in insulin secretion and carbohydrate metabolism [43, 44]. Also, the protective effect of K against obesity could be due to the high intake of fruits and vegetables, as the main sources of K in diet [45]. In summary, more studies are needed to elucidate the exact mechanism of action of dietary K in obesity control.

One of the strengths of the present study was the use of 24-h urinary samples, the most valid and objective indicator to measure total Na and K intakes. However, we only had one 24-h urine excretion per participant, which can cause over/under-estimation of Na and K intake and may be considered a limitation. In addition, although Omron digital scale is a portable measuring tool that enables investigators to assess body composition in a larger population, as we can see in previous studies [46–48], the validity of the device has not been confirmed, thus, the result should be interpreted with caution.

## Conclusion

In conclusion, we found that Na intake among Iranian children and adolescents is high and K intake is lower than recommended levels, with a positive association between Na intake and adiposity. The results provide better insight into the possible relationship between these nutrients and adiposity, emphasizing the necessity of more investigations in this area, which can be resulted in providing a ground for new nutrition policies- such as promoting health interventions and broadening public awareness- to prevent obesity in the future.

## Data Availability

The datasets generated and analyzed during the current study are not publicly available due to institution’s policy but will be available through contacts with the corresponding author on reasonable requests.

## Abbreviations

CVD: Cardiovascular disease;
PAQ: Physical Activity Questionnaire;
BMI: Body mass index;
PBF: Percentage body fat;
LBM: Lean body mass;
FFQ: Food Frequency Questionnaire;
SSBs: Sugar-sweetened beverages;
ANOVA: One-way Analysis of Variance
